# Exploring the role of neuro-immune modulation for psoriasis management: a narrative review of existing evidence and future therapies

**DOI:** 10.1016/j.bbih.2026.101338

**Published:** 2026-08-22

**Authors:** Dragana Savic, Randal A. Serafini, Shen Ning

**Affiliations:** a INIA Biosciences, Inc., Boston, MA, USA; b Venture Development Center, University of Massachusetts Boston, MA, USA; c The Division of Cardiovascular Medicine (CVM), Radcliffe Department of Medicine, University of Oxford, Oxford, UK; d Nash Family Department of Neuroscience, Icahn School of Medicine at Mount Sinai, New York, NY, USA; e Diagnostic and Interventional Radiology, Massachusetts General Hospital, Boston, MA, USA

**Keywords:** Psoriasis, Hypothalamic-pituitary-adrenal axis, Neuro-immune interactions, Vagus nerve stimulation, Splenic focused ultrasound

## Abstract

**Background:**

Psoriasis is a chronic immune-mediated skin disease associated with substantial disease burden and comorbidities including cardiovascular disease, metabolic syndrome, and psychiatric illness. While biologics and small-molecule inhibitors have improved outcomes, disease heterogeneity and the complex drivers of inflammation continue to motivate investigation of complementary therapeutic approaches.

**Major findings:**

Recent work highlights the role of neuro-immune dysregulation in psoriasis. Stress-induced dysregulation of the hypothalamic-pituitary-adrenal axis and increased sympathetic activity have been shown to amplify cytokine production and impair immune regulation. Peripherally, sensory neurons release substance P and CGRP, promoting dendritic cell activation and sustaining Th17-driven inflammation. In contrast, the cholinergic anti-inflammatory reflex, mediated by vagus and splenic nerves through α7 nicotinic acetylcholine receptor signaling, provides an endogenous mechanism to suppress inflammation. Preclinical studies demonstrate that denervation, neuropeptide receptor blockade, vagus nerve stimulation (VNS), and focused ultrasound (fUS) of the splenic nerve attenuate inflammatory immune cell activation and reduce pro-inflammatory cytokine production. Early clinical studies suggest that non-invasive VNS and splenic fUS are safe, feasible, and may reduce systemic inflammatory activity. Additional emerging strategies include neuropeptide receptor blockade, phototherapy-mediated neuroimmune modulation, and lifestyle interventions targeting stress and systemic inflammation.

**Conclusion:**

Psoriasis arises from complex interactions among immune, cutaneous, and neural systems. Growing evidence suggests that neuroimmune pathways may represent therapeutic targets beyond conventional cytokine-directed approaches. While preclinical studies and early clinical investigations support the feasibility of neuromodulatory strategies such as vagus nerve stimulation and splenic focused ultrasound, their efficacy in psoriasis remains to be established. Further mechanistic studies and well-designed clinical trials are needed to define their therapeutic role, optimize treatment parameters, and determine long-term safety and clinical effectiveness.

## Highlights

• Stress-driven HPA axis and neurogenic signaling contribute to psoriatic inflammation.
• CRH signaling, neuropeptides, and cutaneous neuroendocrine pathways promote chronic cutaneous immune activation.
• Vagus nerve stimulation and splenic focused ultrasound target neuroimmune reflex pathways linked to reduced inflammatory signaling.
• UVB phototherapy engages neuroendocrine and immunoregulatory pathways beyond direct immunosuppression.
• Neuroimmune pathways represent potential therapeutic targets beyond conventional cytokine-directed therapies.

## 1 Introduction

Psoriasis is a chronic, immune-mediated inflammatory skin disorder with diverse clinical presentations, including erythrodermic, flexural, guttate, plaque, and pustular subtypes (Raharja et al., 2021). Psoriasis affects millions worldwide and remains a significant public health burden due to its chronic course and associated comorbidities (Rendon and Schäkel, 2019; WHO, 2016). Beyond cutaneous manifestations, psoriasis is associated with cardiovascular, metabolic, and psychiatric comorbidities that substantially contribute to disease burden (Rendon and Schäkel, 2019; WHO, 2016). Several studies have demonstrated enhanced atherosclerosis and an elevated overall risk of cardiovascular disease in psoriasis patients (Masson et al., 2020; Pietrzak et al., 2013). Psoriasis is also associated with components of metabolic syndrome, including obesity, diabetes, and hypertension. Although causality remains debated, these associations are likely multifactorial, reflecting both shared inflammatory pathways and lifestyle factors (Azfar and Gelfand, 2008). In one study, approximately 43% of patients with psoriasis self-reported symptoms of anxiety and 10% self-reported symptoms of depression (Richards et al., 2001). Accordingly, quality-of-life studies have further demonstrated associations between psoriasis and social stigmatization, psychological distress, physical limitations, and employment-related challenges (Christophers, 2001; Kimball et al., 2005).

Despite major advances in systemic therapies, ongoing research continues to explore additional mechanisms that contribute to disease pathogenesis and may provide novel therapeutic opportunities.

**Fig. 1:**
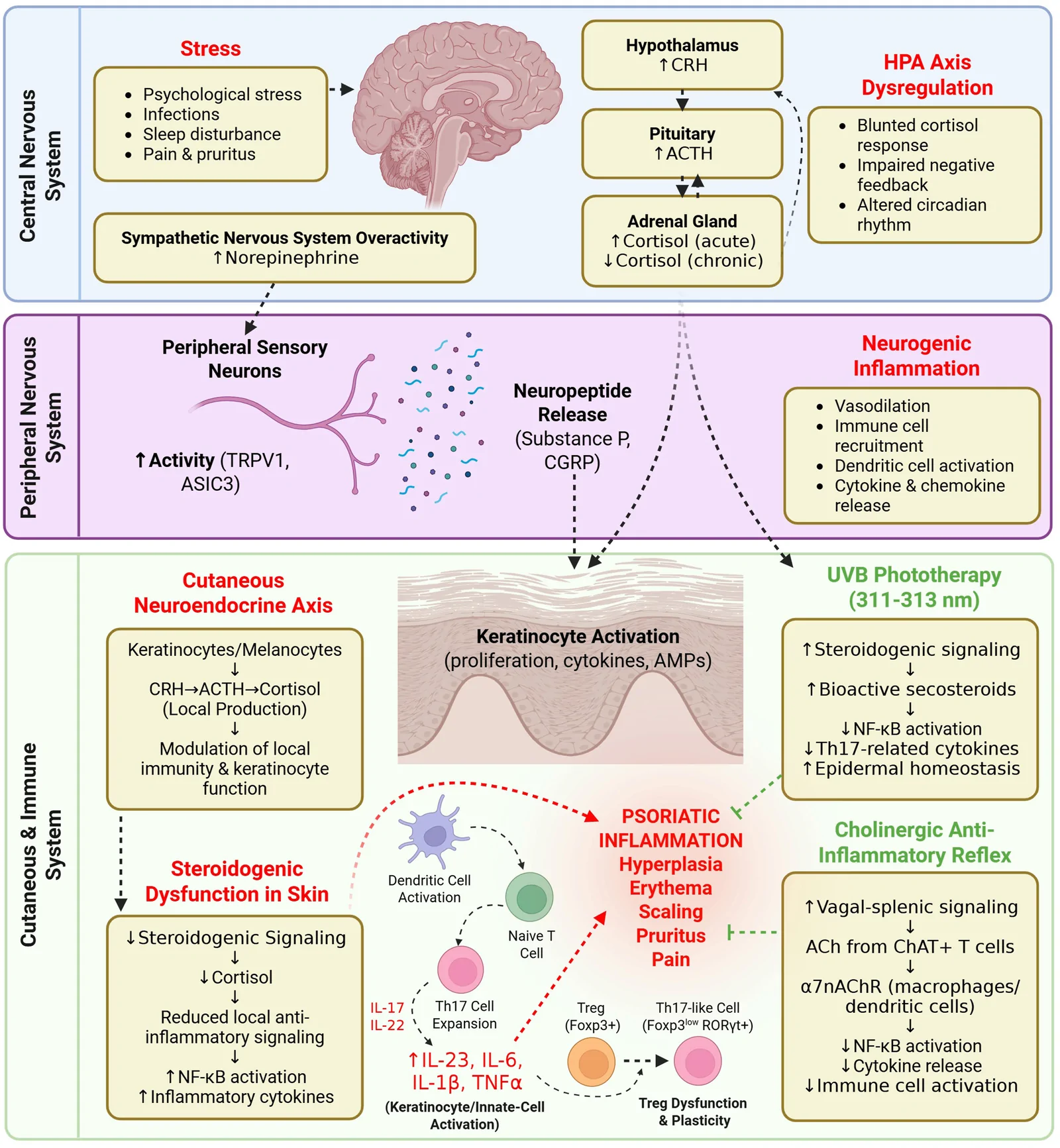
**Neuroimmune mechanisms contributing to psoriasis pathogenesis and potential points of therapeutic modulation. (A) Central neuroendocrine regulation:** Psychological and physiological stressors activate the hypothalamic-pituitary-adrenal (HPA) axis and sympathetic nervous system. Chronic dysregulation of these pathways may alter cortisol signaling, increase catecholamine release, and promote pro-inflammatory immune responses associated with psoriasis. **(B) Peripheral neuroimmune signaling:** Activation of sensory neurons expressing channels such as TRPV1 and ASIC3 promotes release of neuropeptides including substance P (SP) and calcitonin gene-related peptide (CGRP). These mediators contribute to neurogenic inflammation through effects on dendritic cells, keratinocytes, vascular responses, and immune-cell recruitment, thereby supporting IL-23/Th17-driven inflammatory pathways. **(C) Cutaneous neuroendocrine and immunoregulatory pathways:** The skin contains a local neuroendocrine system capable of producing CRH, ACTH, cortisol, and other signaling molecules that influence immune homeostasis. Impaired local steroidogenesis, altered neuroendocrine signaling, and activation of dendritic cell–T-cell inflammatory circuits contribute to psoriatic pathology. Conversely, UVB phototherapy and the cholinergic anti-inflammatory reflex represent immunoregulatory pathways that may attenuate inflammatory signaling and restore aspects of cutaneous homeostasis. While neuroimmune modulation shows promise in preclinical and early clinical studies, its therapeutic role in psoriasis remains under investigation.

This narrative review examines the emerging role of neuro-immune dysregulation in psoriasis pathogenesis, including hypothalamic-pituitary-adrenal axis dysfunction, cutaneous neuroendocrine signaling, sensory neuron-immune interactions, and impaired cholinergic anti-inflammatory reflexes, as summarized in Fig. 1. We further discuss how these pathways may inform the development of novel therapeutic strategies, including bioelectronic and other non-pharmacologic interventions. The literature included in this review was selected in a non-systematic manner to provide a detailed but non-exhaustive overview of the topics above.

## 2 Immunological basis of psoriasis and existing therapies

Psoriasis is marked by chronic inflammation, aberrant keratinocyte proliferation, and dysregulated immune cell differentiation (Rendon and Schäkel, 2019). Psoriatic plaques display acanthosis, or epidermal hyperplasia, alongside dense dermal infiltration by activated dendritic cells, macrophages, T cells and neutrophils (Rendon and Schäkel, 2019). This systemic inflammation arises from a complex imbalance between key dermal cell populations and immune effectors (Chen et al., 2023; Griffiths et al., 2021). Disease initiation is hypothesized to involve the activation of plasmacytoid dendritic cells, which release type 1 interferons (IFN-α and IFN-β) (Chen et al., 2023; Rendon and Schäkel, 2019), potentially triggering downstream activation of myeloid dendritic cells. These cells subsequently release interleukin-23 (IL-23), IL-12, and tumor necrosis factor-α (TNF-α), promoting the differentiation of Th17 and Th1 T helper cell subsets that drive psoriatic inflammation (Chen et al., 2023; Rendon and Schäkel, 2019). Specifically, Th17-associated cytokines, such as IL-17, IL-21, and IL-22, play a central role in driving the sustained proliferation of keratinocytes (Chen et al., 2023). Regulatory T cell (Treg) dysfunction further amplifies this inflammatory cascade by enabling unchecked Th17 and Th1 cell activity. While psoriatic patients have comparable percentages of circulating CD4^+^CD25+Foxp3+ Tregs to healthy controls, these cells exhibit profoundly reduced suppressive function, leading to an altered Th17/Treg balance central to disease pathogenesis (Kanda et al., 2021; Nussbaum et al., 2021). This functional impairment involves dysregulation of the Akt-Foxo1 pathway, causing upregulation of Th1-like transcription factors in Tregs (Li et al., 2019). Under inflammatory conditions enriched with IL-23, IL-6, and IL-21, psoriatic Tregs can downregulate Foxp3, upregulate RORγt, and acquire a Th17-like phenotype, thereby losing suppressive function while actively contributing to disease progression (Bovenschen et al., 2011; Ortega-Mejia et al., 2025). The Th17/Treg ratio is significantly increased in psoriasis and positively correlates with disease severity and Psoriasis Area and Severity Index (PASI) scores, creating a self-perpetuating cycle where impaired Treg suppression allows effector T cells to drive chronic inflammation, which further promotes Treg dysfunction and conversion (Qiao et al., 2023; Shi et al., 2019).

As a result of its central role in psoriasis pathogenesis, the IL-23/Th17 axis has become a major target in therapeutic development (Dand et al., 2020). For example, ustekinumab targets the p40 shared subunit of IL-12 and IL-23, while secukinumab and ixekizumab selectively neutralize IL-17A. More recently, guselkumab and tildrakizumab have been developed to inhibit the p19 subunit of IL-23 (Menter et al., 2019), and while they have demonstrated substantial clinical efficacy, their long-term effectiveness, accessibility, and cost remain important considerations. Additionally, TNF-α inhibitors such as etanercept, infliximab, adalimumab, and certolizumab, have demonstrated robust improvement of PASI scores (Gordon et al., 2012; Gottlieb et al., 2003; Owczarek et al., 2022). Janus kinase-signal transducer and activator of transcription (JAK/STAT) signaling has also been identified as playing a key role in the development of psoriatic disease, particularly psoriatic arthritis, as demonstrated by the clinical efficacy of the JAK inhibitors, tofacitinib and upadacitinib (Rendon and Schäkel, 2019). More recently, deucravacitinib, a selective TYK2 inhibitor within the JAK family, has demonstrated clinical efficacy in moderate-to-severe plaque psoriasis (Armstrong et al., 2023).

Conventional therapies for psoriasis include topical corticosteroids, methotrexate (MTX), cyclosporin A, retinoids, vitamin D analogues, and phototherapy, all of which can improve PASI scores but may be limited by adverse effects, toxicity, contraindications, or long-term safety considerations (Rendon and Schäkel, 2019). Additionally, fumaric acid esters (FAEs), specifically dimethyl fumarate and monomethyl fumarate (DMF/MMF), inhibit the induction of Th1/Th17 responses (Schafer, 2012; Sulaimani et al., 2021). Apremilast, a phosphodiesterase-4 inhibitor that increases cAMP levels, has also been shown to reduce the expression of TNF-α, IFN-γ, and IL-12 and has a broad anti-inflammatory impact on cutaneous cell types (Schafer, 2012).

Despite their effectiveness in reducing disease burden, existing therapeutic options do not fully address the multifactorial nature of psoriatic inflammation, and some patients experience incomplete responses or adverse effects. This has prompted interest in targeting alternative upstream drivers of inflammation, such as neurogenic pathways, which may contribute to disease initiation and persistence through interactions with immune cells, keratinocytes, and vascular elements.

### 2.1 Neuro-immune dysregulation in psoriasis

Neuro-immune interactions in chronic inflammatory diseases are multifaceted. For example, primary sensory neurons, such as nociceptors, can induce cutaneous neurogenic inflammation through the release of neuropeptides that stimulate mast cells, activate keratinocytes, and promote circulating immune cell recruitment. Further, hypothalamic-pituitary-adrenal (HPA) axis dysfunction and sympathetic nervous system overactivity have been associated with Th1/Th17 polarization and leukocyte homing to the skin, while impaired parasympathetic signaling may diminish anti-inflammatory regulation (Ayasse et al., 2020).

### 2.2 HPA axis contributions

The hypothalamic-pituitary-adrenal (HPA) axis and the sympathetic nervous system (SNS) are key pathways through which the central nervous system (CNS) responds to stress. The HPA axis begins with the release of corticotropin-releasing hormone (CRH) from the paraventricular nucleus of the hypothalamus which triggers adrenocorticotropic hormone (ACTH) release from the pituitary, ultimately leading to the secretion of glucocorticoids (GC) (Ayasse et al., 2020). Concurrently, CRH can stimulate noradrenergic neurons and the sympathetic nervous system, promoting the release of norepinephrine (NE) from peripheral nerve terminals and epinephrine from the adrenal medulla (Ayasse et al., 2020). The notion that the CRH system plays a central role in the cutaneous immune response is emphasized by the finding that mast cells are activated by CRH via CRHR1, and psoriatic lesions are characterized by elevated CRH expression, increased sensory nerve density, and increased mast cell count (Theoharides et al., 1998). This response initiates a feedforward loop that perpetuates inflammation and maladaptive cutaneous responses (Theoharides et al., 1998). Additionally, CRH can induce mast cell degranulation, which may act to potentiate a feedforward stress response to the nervous system (Singh et al., 1999).

Although peripheral circulating levels of CRH, ACTH, GC, NE and epinephrine vary throughout the day, elevated baseline glucocorticoids and catecholamines alter antigen-presenting cell function, often reducing IL-12 under acute stress. However, chronic dysregulation can paradoxically promote a shift toward Th1/Th17-mediated inflammation, contributing to elevated IFN-γ, TNF-α, and IL-23 levels. Increased activation of hypothalamic CRH neurons by stress-related regions, including the amygdala and hippocampus, results in sustained activation of the HPA axis and the autonomic nervous system (ANS), ultimately increasing glucocorticoid and inflammatory cytokine levels (Arck et al., 2006; Ayasse et al., 2020; Moynihan et al., 2010). Psychological stress is particularly linked to increased levels of pro-inflammatory mediators IL-1, IL-6 and TNF-α, which are implicated in depressive illness and a range of skin disorders (Ayasse et al., 2020; Hayley et al., 2005). Shared inflammatory mediators, including TNF-α, IL-12, and IL-23, have been implicated in both psoriasis and psychiatric comorbidities (Tong et al., 2022). Interestingly, CRH can also activate noradrenergic pathways in the locus coeruleus, which increases systemic and cutaneous norepinephrine release, ultimately modulating peripheral immune cell and keratinocyte function (Kvetnansky et al., 2009). While initially serving a protective role through β2-adrenergic receptor-mediated immunosuppression, sustained sympathetic activation can exert context-dependent pro-inflammatory effects, including increased epidermal permeability, immune cell recruitment, and inflammatory signaling, thereby exacerbating pruritus and stress-associated disease flares (Nance and Sanders, 2007; Straub and Pongratz, 2013). Sustained CRH release can also adaptively reduce glucocorticoid receptor expression in skin cells, impairing negative feedback and maintaining an inflammatory milieu (Valentino and Van Bockstaele, 2008).

### 2.3 Peripheral nervous system contributions

In addition to the aforementioned effects of chronic CRH activation of peripheral sympathetic drive, increased glucocorticoid and CRH levels can sensitize primary sensory neurons in the dorsal root ganglia (DRGs), thereby promoting the release of pro-inflammatory neuropeptides including substance P (SP) and CGRP (Arck et al., 2006; Slominski et al., 2006). In addition to regulating vascular tone, these peptides can directly activate mast cells, dendritic cells, and keratinocytes, thereby enhancing immune dysregulation in the skin (Theoharides, 2024; Wang and Kim, 2020). Indeed, several studies have found that nociceptors are required for psoriatic inflammation. TRPV1+/Nav1.8+ primary sensory neurons drive the IL-23/IL-17 axis by activating dermal dendritic cells, a pathway that is blunted in knockout mice (Kashem et al., 2015; Riol-Blanco et al., 2014). More recently, the sensory ASIC3 channel was shown to exacerbate imiquimod-induced psoriatic acanthosis and type-17 inflammation, underscoring a broader afferent channel program in cutaneous immunity (Huang et al., 2024).

Denervation studies in the KC-Tie2 model offer causal evidence that neuropeptides are key effectors. Cutting cutaneous nerves reduced CD11c^+^ dendritic cells, CD4^+^ T cells, IL-23, and acanthosis; restoring SP or CGRP signaling reversed discrete parts of the benefit (Ostrowski et al., 2011). Critically, pharmacologic blockade recapitulated denervation: NK1R antagonism (RP67580) lowered CD11c^+^ and CD4^+^ cells, while a CGRP antagonist (CGRP8-37) reduced CD4^+^ cells and acanthosis (Ostrowski et al., 2011). Early pilot studies of NK-1R antagonists demonstrated antipruritic effects, although larger trials failed to meet primary endpoints (Damiani et al., 2022; Pariser et al., 2020). Similarly, botulinum toxin A, which indirectly reduces CGRP and SP release from primary afferent neurons, has shown symptom improvement in small pilot studies (Ghaseminejad-Bandpey et al., 2024). Topical capsaicin, which depletes SP in cutaneous nerves, has also reduced scaling and pruritus in psoriasis patients (Bernstein et al., 1986; Ellis et al., 1993).

**Fig. 2:**
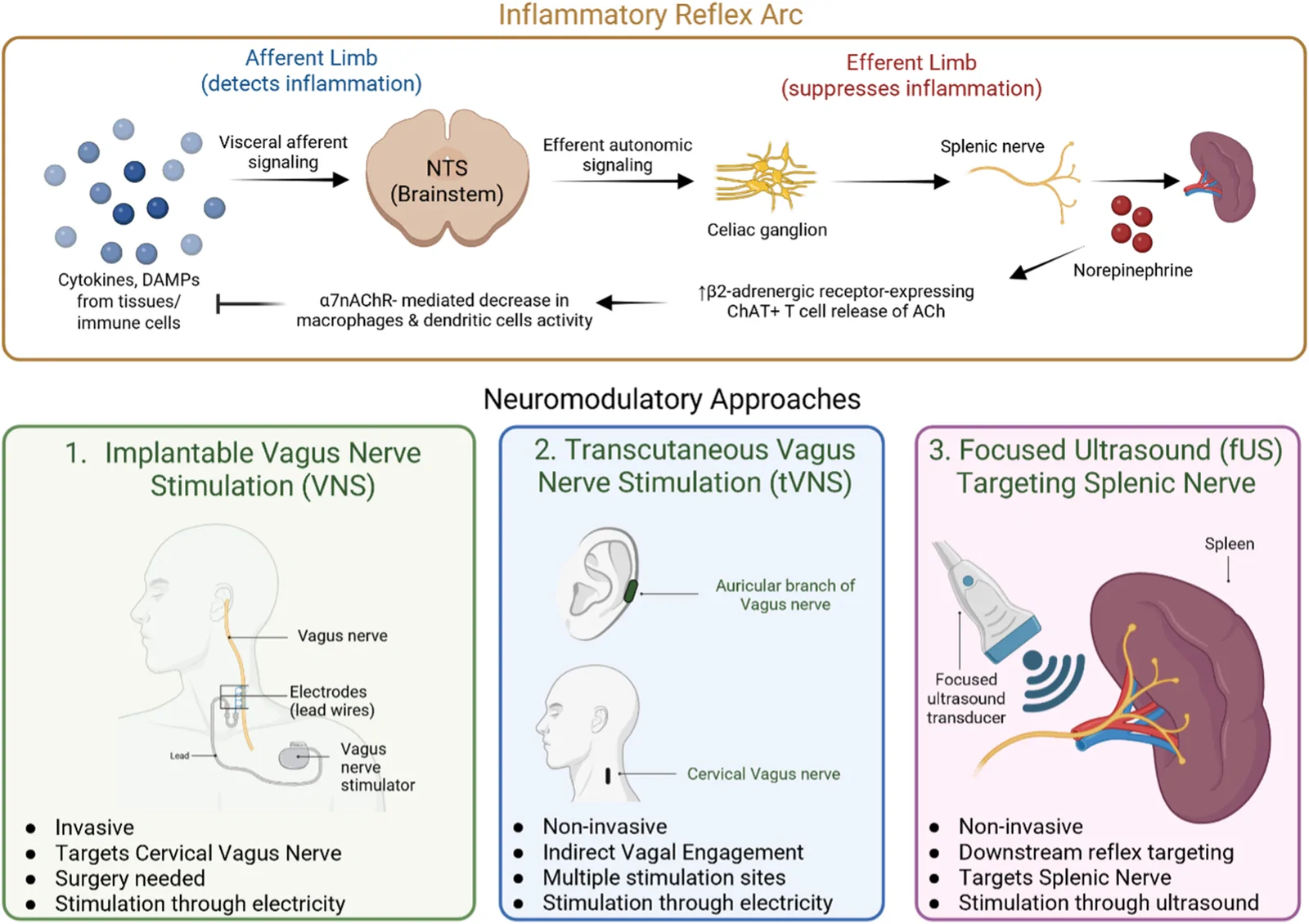
**The cholinergic anti-inflammatory reflex and neuromodulatory approaches for immune regulation. (A) Neuroimmune inflammatory reflex arc:** Peripheral inflammatory signals, including cytokines and damage-associated molecular patterns (DAMPs), are detected by afferent neural pathways and relayed to the nucleus tractus solitarius (NTS) within the brainstem. Efferent autonomic signaling is subsequently transmitted through the celiac ganglion and splenic nerve, resulting in norepinephrine release within the spleen. Activation of β2-adrenergic receptor-expressing ChAT^+^ T cells promotes acetylcholine (ACh) release, which suppresses macrophage and dendritic cell activation through α7 nicotinic acetylcholine receptor (α7nAChR) signaling and reduces inflammatory cytokine production. **(B) Vagus nerve stimulation-based neuromodulation:** Implantable vagus nerve stimulation (VNS) delivers electrical stimulation directly to cervical vagus nerve fibers through a surgically implanted device, whereas transcutaneous vagus nerve stimulation (tVNS) provides non-invasive electrical stimulation through cervical or auricular vagal branches. Both approaches are designed to engage components of the cholinergic anti-inflammatory reflex and have demonstrated immunomodulatory effects in preclinical and clinical studies of inflammatory disease. **(C) Splenic focused ultrasound neuromodulation:** Focused ultrasound (fUS) is a non-invasive approach that targets downstream components of the inflammatory reflex through the splenic neuroimmune interface. Preclinical studies and early human investigations suggest that splenic fUS can modulate inflammatory signaling and reduce pro-inflammatory cytokine production. However, while these findings support the mechanistic plausibility of ultrasound-based neuromodulation, its therapeutic efficacy in psoriasis remains to be established.

Beyond promoting inflammation, primary afferent neurons also participate in feedback mechanisms that regulate immune activity, as described in Fig. 2. Activation of pattern-recognition receptors, including TLR and NLR, induces NF-κB-dependent production of pro-inflammatory cytokines, which can be detected by afferent sensory neurons that form the sensory arc of the inflammatory reflex (Tracey, 2016). These afferent signals are relayed to the brain stem, which in turn activates an efferent cholinergic anti-inflammatory pathway (Tracey, 2009, 2016).

Efferent signals originating in the brainstem ultimately suppress innate immune activation through vagal–splenic neuroimmune pathways and nicotinic acetylcholine receptor subunit α7 (α7nAChR) signaling (Tracey, 2016). The α7nAChR receptor is richly expressed in the skin (Stegemann and Böhm, 2020). Cutaneous cell types such as epidermal keratinocytes, sebocytes and dermal fibroblasts, in addition to immune cells, such as lymphocytes, macrophages and monocytes all express α7nAChR (Stegemann and Böhm, 2020). Specifically, efferent vagal signaling activates the splenic sympathetic pathway, which stimulates ChAT⁺ T cells to release acetylcholine; acetylcholine then binds α7nAChRs on cutaneous and immune cells, suppressing NF-κB activation and pro-inflammatory cytokine production (Tracey, 2016). Selective α7nAChR agonists have demonstrated anti-inflammatory effects in preclinical models and may warrant further investigation in psoriasis (Kelly et al., 2022).

### 2.4 Skin contributions

The skin is increasingly recognized as a neuroendocrine organ that expresses key components of the hypothalamic-pituitary-adrenal (HPA) axis, including CRH, urocortin, POMC-derived peptides, and their corresponding receptors (Slominski et al., 2000; Slominski and Wortsman, 2000). Environmental stressors can activate this neuroendocrine system, leading to the release of hormones, peptides, cytokines, and steroids that modulate immune and neuronal signaling (Slominski et al., 2025). Keratinocytes, melanocytes, fibroblasts, sebocytes, mast cells, and leukocytes can locally release CRH and POMC-derived peptides in response to stress, influencing cutaneous steroidogenic and immune signaling (Slominski et al., 2005, 2007). CRH mediates inflammation induced by lipopolysaccharide in human adult epidermal keratinocytes through activation of NF-κB signaling pathways, demonstrating its dual role in both stress response and inflammatory modulation (Zbytek et al., 2004). Key CRH receptors, CRH-R1 and CRH-R2, are expressed across the dermal and epidermal compartments, with CRH-R1 being the major receptor in humans (Slominski et al., 2006; A. T. Slominski et al., 2013). Alternative splicing of CRHR1 generates receptor isoforms that may alter keratinocyte responsiveness to environmental stressors (Pisarchik and Slominski, 2001; Zmijewski and Slominski, 2009). In psoriasis, dysregulation of the CRH-POMC-steroid axis and local steroidogenesis may contribute to disease pathogenesis. HPA axis dysregulation and neuropeptide signaling may contribute to psoriasis pathogenesis by promoting keratinocyte hyperproliferation and sustaining neurogenic inflammatory loops (Ayasse et al., 2020; Lei et al., 2025; Singh and Taliyan, 2026; A. T. Slominski et al., 2013). Studies have demonstrated upregulation of POMC, CRH receptor type 1, and melanocortin receptors in both lesional and non-lesional psoriatic skin, supporting the importance of the local CRH-POMC system in disease pathogenesis (Galimova et al., 2023; Loite et al., 2013). However, other studies have reported decreased CRH and CRHR1 expression in psoriatic lesions compared to perilesional and normal skin, suggesting that aberrant expression of the CRH/CRHR1 system disturbs local homeostasis and leads to abnormal differentiation and proliferation in keratinocytes (Zhou et al., 2009). Psychological stress exacerbates psoriasis inflammation through HPA axis dysregulation, altering cortisol signaling and systemic immune responses, with evidence of blunted cortisol diurnal rhythm and hypofunctional HPA axis activity in psoriatic patients (Lei et al., 2025; Repousi et al., 2022; Singh and Taliyan, 2026). While data on the specific role of CRHR1 alternative splicing in psoriasis pathophysiology is currently lacking, the established importance of CRHR1 isoform diversity in regulating keratinocyte responses to environmental stress suggests this mechanism warrants investigation in psoriatic disease.

Cutaneous glucocorticoidogenesis also represents a critical component of the local hypothalamic-pituitary-adrenal (HPA) axis in skin, with profound implications for psoriasis pathogenesis. Normal human keratinocytes express the complete enzymatic machinery for *de novo* cortisol synthesis, including CYP11A1, CYP17A1, 3βHSD1, CYP21, and CYP11B1, along with cholesterol transporters StAR and MLN64 that regulate the rate-limiting step of steroidogenesis (Hannen et al., 2011; A. Slominski et al., 2013; R. M. Slominski et al., 2020). These enzymes metabolize cholesterol through pregnenolone and intermediate steroids to produce cortisol in a regulated fashion, creating a local immunosuppressive environment essential for maintaining epidermal homeostasis (Hannen et al., 2011; Phan et al., 2021). In psoriasis, this local steroidogenic system appears dysregulated, representing an additional pathogenic mechanism beyond immune dysregulation (Hannen et al., 2017; Sarkar et al., 2017). Both lesional and non-lesional psoriatic skin demonstrate marked suppression of glucocorticoid biosynthesis and glucocorticoid receptor expression, leading to localized cortisol deficiency (Hannen et al., 2017; Sarkar et al., 2017; Slominski et al., 2017). Metabolomic and transcriptomic profiling has revealed that proinflammatory cytokines actively suppress glucocorticoid biosynthesis in psoriatic epidermis, creating a vicious cycle where glucocorticoid deficiency promotes sustained inflammatory responses and abnormal keratinocyte differentiation (Sarkar et al., 2017). Aberrant expression of StAR and MLN64 in psoriatic skin further indicates dysregulation of the steroidogenic pathway (Hannen et al., 2011). Mouse models with keratinocyte-specific ablation of 11β-hydroxylase (Cyp11b1) demonstrate that long-term glucocorticoid deficiency primes the skin immune system, resulting in spontaneous inflammation and exacerbated psoriasiform responses with decreased regulatory T cells (Phan et al., 2021). This deficient local glucocorticoid feedback on cutaneous immunity contributes to the chronic inflammatory state characteristic of psoriasis (Singh and Taliyan, 2026; Slominski et al., 2017). Topical corticosteroid treatment rapidly normalizes the expression of steroidogenic genes alongside the suppression of inflammation, suggesting that the therapeutic efficacy of topical steroids may partly derive from restoring local steroidogenic capacity in addition to direct exogenous glucocorticoid signaling (Sarkar et al., 2017; R. M. Slominski et al., 2020).

Ultraviolet radiation (UVR) is an important regulator of cutaneous neuroendocrine pathways, with implications for psoriasis pathogenesis and treatment. UVR exposure triggers coordinated neural, hormonal, and immune responses within the skin that contribute to maintenance of local homeostasis (R. M. Slominski et al., 2024). UVB is more effective than UVA at stimulating the production of neuroendocrine signaling molecules, including CRH, urocortin, POMC-derived peptides, and enkephalins, which can exert local and systemic immunomodulatory effects (Slominski et al., 2018). UVB activates the cutaneous HPA axis in a dose- and wavelength-dependent manner, stimulating CRH, POMC-derived peptides, steroidogenic enzyme activity, and local cortisol production (Jozic et al., 2015; Skobowiat et al., 2011; Skobowiat and Slominski, 2015). In psoriasis, phototherapy with narrowband UVB (311-313 nm) represents a cornerstone treatment modality that exploits these photo-neuroendocrine mechanisms to achieve therapeutic benefit (Armstrong and Read, 2020; Griffiths et al., 2021). Narrowband UVB exerts therapeutic effects through multiple mechanisms including induction of keratinocyte and T-cell apoptosis, suppression of inflammatory cytokine production, modulation of dendritic cell activity, and activation of stress-response pathways such as NRF2 and p53 (Addison et al., 2021; Vacharanukrauh et al., 2022; Vieyra-Garcia and Wolf, 2021). Beyond classical vitamin D synthesis, UVB generates CYP11A1-derived vitamin D and lumisterol metabolites with anti-inflammatory, antiproliferative, and antioxidant properties (A. T. Slominski et al., 2024; Slominski et al., 2014, 2021). These CYP11A1-derived vitamin D and lumisterol metabolites regulate multiple pathways involved in psoriasis pathogenesis, promoting antioxidant and DNA repair responses through NRF2 and p53 activation while suppressing NF-κB-mediated inflammatory signaling and downstream cytokine production, including TNF-α, IFN-γ, and IL-17 (Chaiprasongsuk et al., 2020; A. T. Slominski et al., 2024, 2020; Slominski et al., 2014). The therapeutic efficacy of phototherapy in psoriasis likely reflects not only direct immunomodulatory effects on immune cells, but also activation of photo-neuroendocrine pathways that influence local steroidogenesis and inflammatory signaling (Griffiths et al., 2021; Slominski et al., 2018; R. M. Slominski et al., 2024; Vieyra-Garcia and Wolf, 2021). These findings suggest that photo-neuroendocrine pathways and CYP11A1-derived secosteroids warrant further investigation as potential therapeutic targets in inflammatory skin disease (Slominski et al., 2014, 2021).

With regards to cutaneous transcriptomic analyses, a recent study by Gao et al. utilized single cell RNA sequencing of full-thickness healthy and psoriatic human skin samples to demonstrate a pro-inflammatory transcriptional state in epidermal and mesenchymal cells from psoriatic skin, particularly related to IFN and TNF signaling (Gao et al., 2021). Computational analyses predicted that these transcriptional changes could promote dendritic cell activation and inflammation. Additionally, Ma et al. demonstrated that *SFRP2*+ fibroblasts within the papillary dermis can amplify pro-inflammatory signaling in psoriasis through CCL and CXCL signaling that activates myeloid cells, dendritic cells, T-cells, and keratinocytes (Ma et al., 2023). Interestingly, short-term treatment with risankizumab (IL-23 blockade) can reduce the abundance of pro-inflammatory fibroblast populations in psoriatic tissue (Francis et al., 2024).

Although current therapies primarily target inflammatory pathways, increasing attention has focused on approaches that modulate neuroimmune signaling through complementary mechanisms. This has driven interest in non-invasive neuromodulation approaches, including vagus nerve stimulation as a means of regulating immune activity.

## 3 Therapeutic activation of the cholinergic anti-inflammatory reflex

### 3.1 Vagus nerve stimulation

Two non-pharmacologic approaches that leverage the cholinergic anti-inflammatory reflex, vagus nerve stimulation (VNS) and splenic ultrasound (US) (seeFig. 2), are being investigated for inflammatory disease and may have relevance to psoriasis. The current understanding of this reflex pathway, which serves as the foundation for the development of these technologies, is largely based on the early work of Kevin Tracey and colleagues, who made the seminal observation that acetylcholine (ACh) and nicotine reduced the inflammatory responses of macrophages, which ultimately led to the discovery of the inflammatory reflex. Tracey and colleagues hypothesized that ACh mediated the anti-inflammatory effects via a nicotinic receptor (nAChR), as opposed to a muscarinic receptor (mAChR) (Borovikova et al., 2000). Noting that the vagus nerve and its principal neurotransmitter, acetylcholine, are key regulators of the parasympathetic nervous system, Tracey and colleagues transected the vagus nerve (bilateral cervical vagotomy) in rats subjected to LPS-induced endotoxemia (Borovikova et al., 2000). The vagal transection led to an increased systemic inflammatory response, which presented as earlier onset of shock and increased levels of TNF-α (Borovikova et al., 2000). However, electrical stimulation of the distal portion of the transected vagus nerve remarkably attenuated this response and prevented the development of shock (Borovikova et al., 2000). Since 2000, VNS has been investigated in numerous inflammatory conditions, including rheumatoid arthritis (RA), inflammatory bowel disease, peritonitis, and endotoxemia (Caravaca et al., 2022a, 2022b; Koopman et al., 2016; Peterson et al., 2024).

In humans, a recent clinical trial investigating the safety and efficacy of a neuroimmune modulation device implanted in human patients on the left cervical vagus nerve within the carotid sheath found that there were minimal serious adverse events (Peterson et al., 2024). However, some patients experienced the surgery-related adverse event of vocal cord paresis and voice hoarseness, which fully or partially resolved after vocal cord augmentation injections and speech therapy (Peterson et al., 2024). Furthermore, careful parameter optimization is required to minimize off-target effects such as bradycardia (Peterson et al., 2024). Of note, this same device was recently approved by the FDA for the treatment of moderate-to-severe rheumatoid arthritis after the technology demonstrated clinically meaningful ACR20 responses and reduced bone erosion, with minimal serious adverse events (Tesser et al., 2024).

Transcutaneous vagus nerve stimulation has been implemented to reduce any surgical burden associated with implantable technologies. In a small clinical study, Brock et al. recently found that handheld cervical transcutaneous vagus nerve stimulation (t-VNS) resulted in reduced clinical disease activity and decreased C-reactive protein blood levels in patients with psoriatic arthritis (Brock et al., 2021). Cervical t-VNS treatment in one patient with recurrent non-migraine headaches and facial seborrheic dermatitis resulted in a reduction of both the headache frequency and severity, as well as the facial dermatitis (Nahm et al., 2025). In another small study, Cuberos Paredes et al. found that auricular t-VNS was capable of reducing stimulated cortisol levels in a mental stress test, suggesting possible utility in attenuating the HPA axis (Cuberos Paredes et al., 2025). In a proof-of-concept study of pediatric inflammatory bowel disease, auricular t-VNS was associated with clinical remission in a subset of patients and demonstrated a favorable safety profile (Sahn et al., 2023). Indeed, a systematic review by Redgrave et al. further supports the strong safety profile of transcutaneous vagus nerve stimulation (Redgrave et al., 2018).

While direct studies in cutaneous psoriasis are limited, these findings in RA, IBD, and psoriatic arthritis underscore the relevance of vagus-mediated modulation given the shared IL-23/Th17 and TNF-α inflammatory axes. However, stimulation of non-target neural pathways, such as those impacting cardiac activity, may be minimized through selective targeting of downstream components of the cholinergic anti-inflammatory reflex, such as the splenic nerve (Cotero et al., 2019).

### 3.2 Focused ultrasound modulation of the Splenic Nerve

The splenic nerve plays a key role in the cholinergic anti-inflammatory reflex. Several studies have shown that splenectomy and transection of the splenic nerve abolished the anti-inflammatory effects of vagus nerve stimulation (VNS) and lead to increased TNF-α release in response to endotoxemia and sepsis (Huston et al., 2006; Rosas-Ballina et al., 2008; Vida et al., 2011). The essential role of the splenic nerve is thought to be related to its ability to release NE in proximity to choline acetyltransferase-positive (ChAT+) β2-adrenergic-receptor-positive (β2AR+) T cells, which release ACh and can modulate the macrophage immune response and cytokine secretion (Rosas-Ballina et al., 2008); however, the precise nature of this neuro-immune communication remains debated. Focused ultrasound (fUS) has recently emerged as a non-invasive approach for targeting the splenic nerve (Cotero et al., 2020).

The Okusa group analyzed the impact of the splenic cholinergic anti-inflammatory pathway on renal ischemia-reperfusion injury in mice by utilizing splenic nerve-targeting fUS prior to renal ischemia induction (Gigliotti et al., 2013). Twenty-four hours post-reperfusion, US-treated mice exhibited preserved kidney morphology and function (Gigliotti et al., 2013). It was found that compared to sham-treated mice, US-treated mice had reduced accumulation of CD11b + Ly6G^high^ neutrophils and CD11b + F4/80^high^ myeloid cells in kidney tissue (Gigliotti et al., 2013). However, blockade or reduction of the α7nAChR receptor negated the beneficial effect of US, further implicating the role of the cholinergic anti-inflammatory pathway (Gigliotti et al., 2013).

Cotero and colleagues utilized fUS-mediated neuromodulation of the splenic pathway and monitored the response of splenic sympathetic nerve terminals, intermediary T-cells, and macrophages that release cytokines, when mice were challenged with LPS-induced acute inflammation (Cotero et al., 2019). fUS stimulation increased splenic acetylcholine and norepinephrine levels while reducing systemic and splenic TNF-α production, consistent with activation of the cholinergic anti-inflammatory reflex (Cotero et al., 2019). The group systematically demonstrated the requirement of functional T cells, T cells that produce acetylcholine, and cytokine generating macrophages for this response by conducting experiments in depletion and knockout models (Cotero et al., 2019). Although the wild-type, immune-intact C57 mouse population showed a reduction in splenic TNF-α when treated with US, the depletion models showed no reduction in splenic TNF-α compared to the sham (Cotero et al., 2019). This study further linked splenic nerve stimulation to inflammatory reflex pathways relevant to cytokine regulation.

In a companion study to Cotero and colleagues, Zachs and colleagues sought to establish specific fUS stimulation parameters and specific target cell types for the treatment of polyarthritis in mice (Zachs et al., 2019). Using fUS parameters informed by prior studies, the investigators evaluated the therapeutic effects of splenic neuromodulation in a murine model of inflammatory polyarthritis (Gigliotti et al., 2013; Zachs et al., 2019). Splenic fUS reduced polyarthritis-induced joint swelling and was associated with differential gene expression in B and T cells, supporting immunomodulatory effects on both innate and adaptive immune pathways (Zachs et al., 2019).

Findings from animal models have prompted investigation of splenic fUS in human inflammatory disease. In a preliminary human study, splenic fUS was associated with reduced *ex vivo* TNF-α production following LPS stimulation of whole blood, providing early evidence that non-invasive ultrasound neuromodulation can modulate inflammatory responses in humans (Graham et al., 2020). In a subsequent study of healthy volunteers, splenic fUS significantly reduced *ex vivo* TNF-α production following endotoxin challenge, particularly when targeted to the splenic hilum, while demonstrating a favorable safety profile with no significant adverse events (Zanos et al., 2023).

To better understand how splenic fUS may engage neuroimmune pathways, studies have investigated the effects of ultrasound on neuronal activity at the cellular level. An *in vitro* study by Vasan et al. found that fUS can induce small mechanical deformations in neuronal membranes that may influence cellular signaling (Vasan et al., 2022). These mechanical effects have been linked to activation of mechanosensitive ion channels, including TRPP1/2, TRPC1, TRAAK and Piezo1 (Qiu et al., 2019; Sorum et al., 2021; Yoo et al., 2022). *In vitro*, modulation of these channels can alter neuronal excitability, while genetic or pharmacologic disruption of mechanosensitive pathways attenuates ultrasound-induced responses (Yoo et al., 2022). Single-cell studies, in both 2D culture format and 3D collagen cultures, have found little evidence that clinically relevant ultrasound parameters produce cellular injury through thermal effects, cavitation, or membrane damage (Sorum et al., 2021; Yoo et al., 2022).

Available preclinical and early clinical studies suggest a favorable safety profile for fUS neuromodulation, with no significant effects on measured biochemical or hematological parameters. Its demonstrated effects on inflammatory mediators, including TNF, support further investigation of splenic ultrasound as a non-pharmacologic immunomodulatory strategy. Although evidence in psoriasis remains indirect, targeted clinical trials could establish whether this approach can complement existing therapeutic strategies and provide clinical benefit in psoriatic disease.

## 4 Additional novel approaches for psoriasis treatment

Additional medtech-based strategies are under exploration for immune-mediated dermatologic conditions. Many of these approaches aim to improve treatment precision, convenience, or tolerability while preserving therapeutic efficacy. For example, wearable phototherapy devices, including targeted narrow-band UVB patches, are being developed to deliver localized treatment with improved safety profiles compared to full-body phototherapy. In a small study, Kemény et al. found that a UVB-LED phototherapeutic device effectively reversed psoriatic plaques (Kemény et al., 2010). Additionally, microneedle arrays are being studied for transdermal delivery of biologics and small molecules, offering pain-free administration and potential for dose sparing. In a pre-clinical study, Du et al. found that a methotrexate-loaded hyaluronic acid microneedle patch more effectively reversed psoriasis-like inflammation in mice than equivalent oral dosing, while reducing systemic toxicity (Du et al., 2019). However, clinical-stage work on microneedle drug administration has been limited to human *exvivo* skin preparations (Ruel et al., 2025; Vora et al., 2022) – therefore future studies will be necessary to determine if similar therapeutic efficacy can be observed in psoriasis patients. Non-invasive electrical stimulation modalities, such as transcutaneous electrical nerve stimulation (TENS), have been piloted for pruritus reduction (Zuo et al., 2024), although evidence remains limited and current studies primarily address symptom burden rather than disease pathogenesis. Advances in digital health, including smartphone-integrated apps/imaging and artificial intelligence algorithms, are enabling real-time disease monitoring, individualized treatment adjustment, and mental health support (Fortune et al., 2022; Goessinger et al., 2024), while also improving medication adherence (Svendsen et al., 2018). While early, these approaches exemplify how novel device-based and digital innovations could complement systemic and topical therapies to improve long-term disease management.

In addition to device-based innovations, accessible lifestyle interventions targeting systemic inflammation and neuroimmune balance represent promising adjunctive strategies for psoriasis management. Physical activity has documented anti-inflammatory effects and may positively influence psoriasis through metabolic, immunologic, and psychosocial mechanisms (Balato et al., 2015; Ko et al., 2019). Exercise has been associated with increased regulatory immune activity and release of anti-inflammatory myokines, which can promote IL-10 production and suppress pro-inflammatory signaling in a context-dependent manner (Sharif et al., 2018). Stress reduction techniques, including meditation and guided imagery, have shown modest efficacy as adjunctive therapies, with some studies suggesting that meditation may enhance treatment response during phototherapy (Gamret et al., 2018). The Joint AAD-NPF Guidelines acknowledge that meditation can have a positive impact on psoriasis symptom severity and may be discussed as adjunctive therapy with interested patients (Elmets et al., 2021). Dietary modification also influences disease activity, with the strongest evidence supporting hypocaloric diets for weight reduction in overweight and obese patients, as adipose tissue produces inflammatory adipokines that propagate both psoriasis and its metabolic comorbidities (Elmets et al., 2021; Ford et al., 2018). The Mediterranean diet, emphasizing whole fruits, vegetables, grains, and healthy fats, offers anti-inflammatory and cardiovascular benefits suitable for psoriasis patients, while gluten-free diets may benefit the subset of patients with serologic markers of gluten sensitivity (Elmets et al., 2021; Ford et al., 2018). Patients should also be counseled to avoid excessive alcohol consumption, which increases TNF-α expression, and smoking, which induces free radical production and promotes inflammation (Ko et al., 2019). These accessible interventions, including physical activity, stress reduction, and dietary optimization, may complement pharmacologic and device-based therapies by addressing factors that contribute to systemic inflammation and disease burden.

## 5 Conclusion

Psoriasis is a chronic inflammatory disease characterized by complex interactions among immune, cutaneous, and neural systems. Although current therapies have substantially improved outcomes, ongoing research continues to explore additional mechanisms that contribute to disease pathogenesis and may provide complementary therapeutic opportunities. Among the neuroimmune pathways implicated in psoriasis, the cholinergic anti-inflammatory reflex has emerged as a potential target for therapeutic modulation. Preclinical and early clinical studies suggest that immune activity can be influenced through modulation of key components of this reflex pathway. Future studies investigating neuroimmune signaling pathways, including NF-κB regulation, α7nAChR signaling, and bioelectronic approaches, may further clarify the role of neural-immune interactions in inflammatory disease. Additional mechanistic studies and well-designed clinical trials will be necessary to identify relevant target cell populations, optimize treatment parameters, and establish the safety, efficacy, and reproducibility of these emerging approaches in psoriasis.

## CRediT authorship contribution statement

**Dragana Savic:** Writing – original draft, Project administration, Conceptualization. **Randal A. Serafini:** Writing – review & editing, Visualization, Project administration, Conceptualization. **Shen Ning:** Writing – original draft, Project administration, Conceptualization.

## Funding

No funding sources were used for this review.

## Declaration of competing interests

The authors declare the following financial interests/personal relationships which may be considered as potential competing interests:Shen Ning reports a relationship with INIA Biosciences that includes: equity or stocks. Dragana Savic reports a relationship with INIA Biosciences that includes: employment and equity or stocks. Randal A. Serafini reports a relationship with INIA Biosciences that includes: consulting or advisory. If there are other authors, they declare that they have no known competing financial interests or personal relationships that could have appeared to influence the work reported in this paper.

## Data Availability

No data was used for the research described in the article.
